# Gene delivery to neurons in the auditory brainstem of barn owls using standard recombinant adeno-associated virus vectors

**DOI:** 10.1016/j.crneur.2020.100001

**Published:** 2020-08-06

**Authors:** Nadine Thiele, K. Jannis Hildebrandt, Christine Köppl

**Affiliations:** a Department of Neuroscience, School of Medicine and Health Sciences, Carl von Ossietzky University Oldenburg, 26129, Oldenburg, Germany; b Cluster of Excellence “Hearing4all” and Research Center Neurosensory Science, Carl von Ossietzky University Oldenburg, 26129, Oldenburg, Germany

**Keywords:** AAV, Bird, Avian, Optogenetics

## Abstract

Recombinant adeno-associated virus (rAAV) vectors are a commonly used tool for gene delivery. There is a large choice of different serotypes whose transduction efficiency varies for different animal species. In this study, three rAAV vectors were tested for transduction efficiency in the auditory brainstem of adult barn owls (*Tyto alba*) which are not standard laboratory animals. Injections with rAAV serotypes 2/1 and 2/5 resulted in reliable expression in various nuclei of the auditory brainstem of barn owls. Both vectors showed evidence of being spread by axonal transport. However, only rAAV2/5 also showed expression in regions far distant from the injection site, suggesting long-range axonal transport in connections along the auditory pathway. In contrast, injections with rAAV2/9 resulted in no expression. Our results demonstrate for the first time that commercially available rAAV vectors can be used for reliable gene expression in the barn owl auditory brainstem and pave the way toward optogenetic manipulation of neural activity in this important animal species in neuroethology and auditory physiology.

## Highlights

• Standard rAAV vectors effectively mediate gene expression in the barn-owl brain.
• rAAV2/1 was best suited for confined expression around the targeted injection site.
• Due to more axonal transport, rAAV2/5 resulted in wider spread of expressed genes.
• rAAV-mediated expression is expected to remain stable for an extended period of time.
• rAAV expression and stability are excellent prerequisites for optogenetic experiments.

## Graphical abstract

**Figure:**
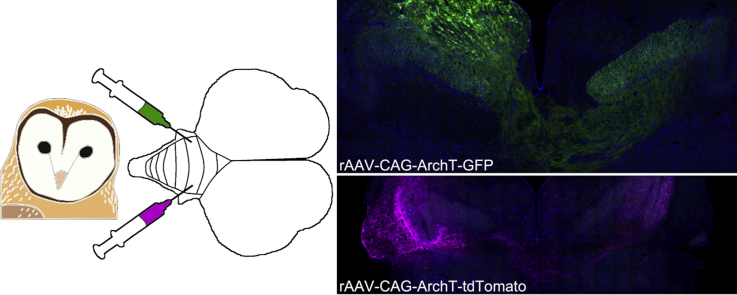

## 1 Introduction

In recent years, recombinant adeno-associated virus (rAAV) vectors have become a commonly used tool in optogenetics. They enable targeted expression of genes for light-sensitive proteins, to manipulate the cells’ activity using light stimulation under controlled experimental conditions (Boyden et al., 2005; Yizhar et al., 2011). The aim of the current study was to identify reliable rAAV serotypes for such gene transduction in the barn-owl brainstem for future optogenetic experiments. There is a long history of insightful research on barn owls, in auditory physiology and neuroethology, that continues today (Carr & Pena, 2016; Konishi, 2003; Payne, 1971; Pena et al., 2019). Adding optogenetics as a state-of-the art tool will open up new possibilities for precise experimental manipulation. Our ultimate aim is to inhibit neural activity in various nuclei of the auditory brainstem, such as nucleus laminaris (NL), which is involved in the binaural processing of interaural time differences (ITD) (Carr & Boudreau, 1993; Carr et al., 2015). To this end, three different rAAV serotypes were injected into the brainstem of barn owls, with different injection volumes and expression times, and their transduction efficiencies were evaluated.

Adeno-associated viruses (AAVs) are small parvoviruses (∼22 ​nm) containing single-stranded DNA (∼4.7 ​kb) (Goncalves, 2005; Xiao et al., 1997). Replication-defective recombinant adeno-associated virus (rAAV) vectors have become important tools used for gene delivery in a wide range of different species and tissues. Due to the lack of viral genes, rAAV injection causes a reduced immune reaction (Xiao et al., 1997), except when repeatedly administered at short intervals (Mastakov et al., 2002; Mendoza et al., 2017). Furthermore, rAAV vectors are typically classed as genetically modified organisms of the lowest risk category and thus do not require expensive lab space that satisfies high-level safety standards, as do, e.g., lentiviral vectors. Several studies have compared different AAV serotypes for transduction efficiency. Experiments with mice demonstrated different expression levels, onset times and different infection rates for a range of organs and tissues, after injection into the tail vein or brain, respectively (Aschauer et al., 2013; Zincarelli et al., 2008). Similarly, in non-standard animal species, AAV-mediated gene expression was successful in, e.g., the brains of cats (Rockwell et al., 2015), gerbils (Keplinger et al., 2018) and the retina of non-human primates (Stieger et al., 2006; Watakabe et al., 2015). This also extends to non-mammalian species like the zebra finch, where expression in brain tissue was also successful (Heston & White, 2017; Roberts et al., 2012). Serotype-specific tropism can be improved by using so-called pseudotyping. Tissue specificity of AAVs is determined by the viral capsid. Therefore, expression results may be improved by cross-packaging the genome of one serotype (in general AAV2) containing the gene of interest, with the capsid of another serotype (Burger et al., 2004; Rabinowitz et al., 2002) that is tailored to the target tissue. Targeting can be further improved with cell-specific promoters. In the case of brain transduction with rAAV, the cytomegalovirus (CMV) and a hybrid construct consisting of CMV early enhancer and chicken beta actin (CAG) promoter are common choices for neuronal expression (Fitzsimons et al., 2002; Niwa et al., 1991).

Many experiments have shown that rAAV serotypes can undergo axonal transport within the central nervous system (CNS). Depending on the serotype, either retrograde or anterograde transport was observed, in mice (Aschauer et al., 2013; Rothermel et al., 2013), rats (McFarland et al., 2009; Salegio et al., 2013), non-human primate brains (Salegio et al., 2013), and zebra-finch brains (Heston & White, 2017). Bidirectional transport is also possible, as shown in mice (Castle et al., 2014; Cearley & Wolfe, 2007) and rats (Castle et al., 2014). Axonal transport increases the spatial reach of an injection and, depending on the application, may be either undesirable, or even used to advantage. In summary, it is crucial to choose a suitable serotype for the specific experimental design, because of large variability in transduction efficiency of the different rAAV serotypes.

In the barn owl, injection of rAAV2/1 and rAAV2/5 resulted in reliable expression in nucleus laminaris and nucleus magnocellularis (NM), whereas we were not able to achieve expression using rAAV2/9. Furthermore, with rAAV2/5, axonal transport to distant auditory nuclei in the brainstem and midbrain occurred. Our results show that commercially available rAAV vector solutions for gene delivery are useful in a non-standard animal species like the barn owl.

## 2 Material and methods

### 2.1 Experimental animals and preparation

We report data from 11 adult European barn owls (*Tyto alba*) of both sexes and aged between one and five years.

Animals were deprived of food for ∼12 ​h before the initiation of anesthesia and were anesthetized with an initial dose of 10 ​mg/kg ketamine hydrochloride (aniMedica, LEVISTO company) and 3 ​mg/kg xylazine hydrochloride (Dechra Veterinary Products), via intramuscular injection. Maintenance doses of 1.6–5 ​mg/kg ketamine and 0.6–1.8 ​mg/kg xylazine were given as needed, typically every 30 ​min. Before the surgery, the animals received a single dose of 0.05 ​mg/kg buprenorphine (Bayer Vital), an analgesic used for pain treatment. After each experiment, the owl received a single dose of 0.02 ​mg/kg meloxicam (Boehringer Ingelheim Vetmedica), a nonsteroidal anti-inflammatory drug, for the recovery phase. EKG recording was used for monitoring vital signs and a heat blanket (Harvard Apparatus) to maintain the body temperature at 39 ​°C.

All procedures took place in a double-walled chamber (model 1203A, Industrial Acoustics). During the experiments, the animal's head was held using a custom-built beak holder. In addition, a small metal plate connected to the stereotaxic system (Kopf Instruments), was fixed to the skull using dental cement (Paladur, Heraeus Kulzer). A reference point was marked on the midline between the ear bars and a small skull opening was made 1–5 ​mm caudal and 1–4 ​mm lateral relative to the reference. The dura mater was cut before inserting the recording electrode or injection needle. At the conclusion of the experiment, the exposed brain area was overlain with a gelatin sponge (Gelastypt, Sanofi-Aventis) and covered with dental cement before the skin was sutured. The experimental procedure was carried out on both sides of the brainstem, with one week of recovery in between. After one to five weeks expression time, the animals were killed by an overdose of sodium pentobarbital (∼100 ​mg/kg), perfused transcardially with 4% paraformaldehyde in phosphate-buffered saline (PBS) and the brains were analyzed for expression patterns.

### 2.2 AAV vectors and injections

Three commercially available rAAV vectors (Boyden lab, MIT, provided by UNC Vector Core, University of North Carolina, USA) were injected into the brainstem of barn owls. rAAV2/1-CAG-ArchT-GFP (2.1 ​× ​10^12^ particles/ml), rAAV2/5-CAG-ArchT-tdTomato (3.1 ​× ​10^12^ particles/ml) and rAAV2/9-CAG-ArchT-GFP (3.0 ​× ​10^12^ particles/ml). These rAAV vectors have different serotypes, containing the genome of AAV2 and the capsid of serotype 1, 5 or 9. All vectors contained the same light-sensitive archaerhodopsin from *Halorubrum* strain TP009 (ArchT) for inhibition (Han et al., 2011) and the ubiquitous neuronal promoter CAG, which is a hybrid promoter with optimized expression results (Halbert et al., 2007).

**Fig. 1:**
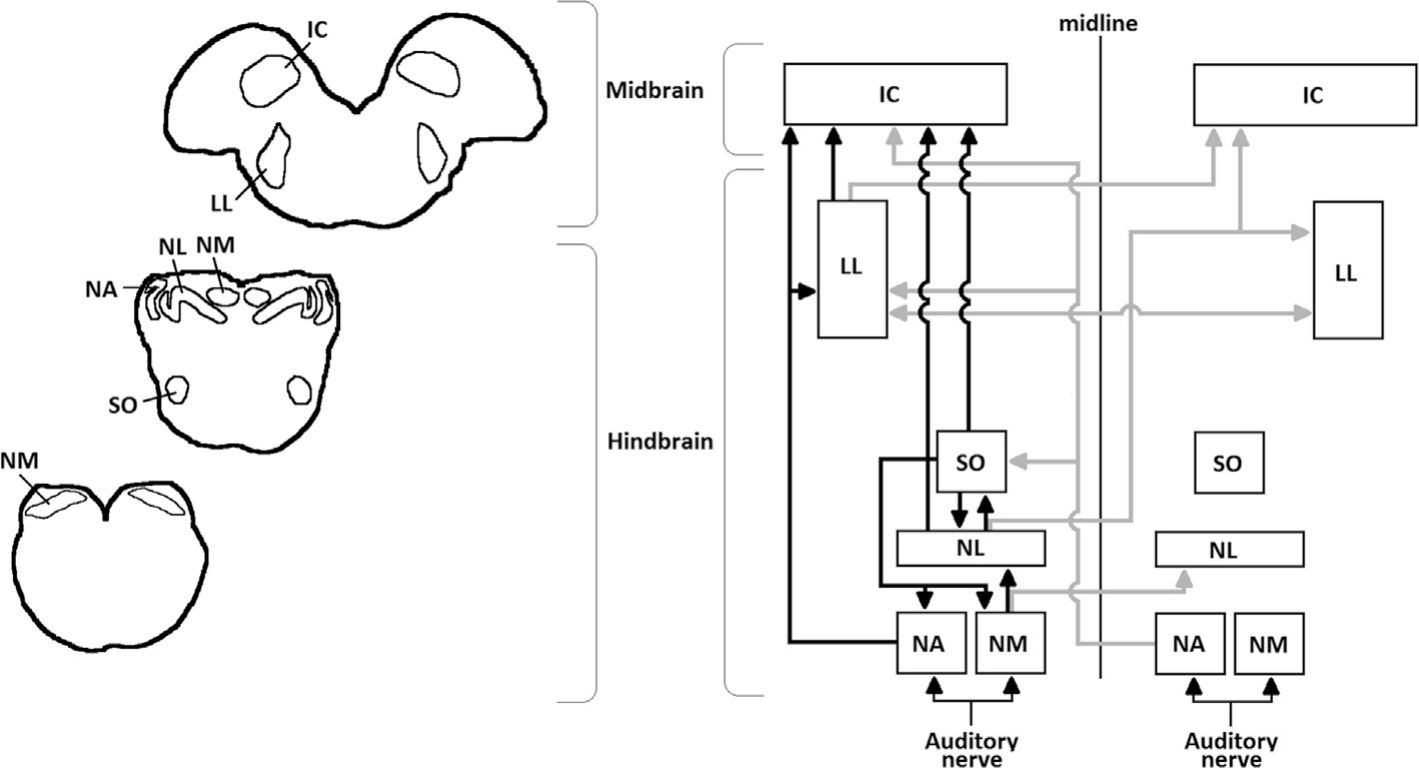
**Overview of avian auditory pathway**. **Left:** Outlines of 3 representative cross sections of the barn owl's brainstem, arranged from caudal (lower left) to rostral (upper right). Nucleus magnocellularis (NM), n. laminaris (NL), n. angularis (NA), superior olive (SO), lateral lemniscus (LL) and inferior colliculus (IC) are also outlined. **Right:** Schematic wiring diagram of the avian auditory pathway (adapted from Konishi, 2000). Ipsilateral connections are shown in black and contralateral connections in grey. The auditory nerve innervates two cochlear nuclei: NM, which sends projections to ipsi- and contralateral NL, and NA, which innervates the SO in the brainstem and LL and IC in the midbrain. The SO sends feedback projections to all 3 lower-level auditory brainstem nuclei and forward projections to the IC. The NL has connections to the SO, LL and IC. The LL connects to the IC and to the contralateral LL.

The intended injection sites in the brainstem can only be reached after first penetrating between 7 and 10 ​mm of cerebellum. Furthermore, there is no standard stereotaxic brain atlas for the barn owl. Therefore, to identify a target region for the virus injection, we first carried out electrophysiological recordings with tungsten microelectrodes while presenting auditory stimuli through a custom-made headphone system. The target regions for injection and expression were nucleus laminaris (NL) or nucleus magnocellularis (NM). Both nuclei are part of the auditory pathway, occupy overlapping regions of the brainstem, and are directly connected (NM projects bilaterally to NL (Carr & Konishi, 1990); see Fig. 1 for an overview).

After establishing a suitable set of stereotaxic coordinates, the recording electrode was replaced by the injection needle. Different volumes of the virus vector solution were injected into the brainstem target site using a Hamilton syringe (1701 RN, 10 ​μl syringe, Hamilton) with a 33G needle attached (Small Hub RN Needle, point style 4, Hamilton), and connected to a Micro4 controller (World Precision Instruments) for pressure injection. Using a manually operated micromanipulator, the needle was slowly lowered through the cerebellum and into the brainstem. Once the desired depth (between 8.5 and 11 ​mm) had been reached, 1–4 ​μl were injected at a rate of 150 ​nl/min (see Table 1 for more details). The needle was then left in place for a further 10 ​min, before slowly retracting it again.

**Table 1:** **Parameters and outcomes of rAAV vector injections in the barn owl**. Column 1 numbers the cases consecutively, for easy reference in the main text and figure legends. Note that these are not animal IDs, which are listed in column 2. Also note that cases of off-target injections are listed first, then the on-target injections. Column 3 shows the viral vector used, column 4 the injected volume, and column 5 the expression time after injection. Column 6 lists the brainstem side of injection, and column 7 the estimated location of the injection center. Column 8 lists all regions where expression of the fluorescent tag was observed, together with some detail on the expression pattern. Column 9 details the putative axonal transport routes. Finally, column 10 refers to the relevant figure, if applicable.

	Case	Animal ID	Vector	Volume (μl)	Exp. Time (weeks)	Side of Injection	Center of Injection Site	Expression Regions	Axonal Transport	Figure
**Off-Target**	#1	1	rAAV2/1	1	3	left	not found, likely more rostral than evaluated	NM: cell bodies sparsely spread over entire extent of both NM; NL: sparse fibers over entire extent of both NL		
	#2	1	rAAV2/1	1	4	right	not found, likely more rostral than evaluated	NM: cell bodies sparsely spread over entire extent of both NM; NL: sparse fibers over entire extent of both NL		
	#3	4	rAAV2/1	2	3	right	not found, likely more rostral than evaluated	brainstem: sparse fiber fragments		
	#4	6	rAAV2/1	2	3	right	cerebellum	cerebellum: fibers and cell bodies, both ipsi- and contralateral		
	#5	8	rAAV2/1	2	3	right	cerebellum	cerebellum: few fibers ipsilateral; NL: some fibers inside and around ipsilateral NL		
	#6	5	rAAV2/1	2	5	right	cerebellum	NM: few cell bodies in both ipsi- and contralateral NM; NL: few fibers in and around ipsilateral NL; cerebellum: sparse cell bodies and fibers in ventral part, above brainstem		
	#7	4	rAAV2/5	2	4	left	not found, likely more rostral than evaluated	NL: few fibers and cell bodies in the lateral part of contralateral NL; SO: few fibers and cell bodies in dorsal part of contralateral SO		
	#8	6	rAAV2/5	2	4	left	rostral brainstem, towards midbrain	NL: few cell bodies and fibers in lateral part of contralateral NL; NA: few cell bodies in contralateral NA; SO: fibers and cell bodies spread over entire contralateral SO; LL: cell bodies and fibers in contra- and ipsilateral nuclei, ipsilateral over entire extent, contralateral mainly dorsal nucleus	probably retrograde vector transport to NL, NA, SO and LL	2A; 2B
	#9	7	rAAV2/5	4	1	left	rostral brainstem, towards midbrain	cerebellum: few cell bodies and fibers in ventral part of ipsilateral cerebellum; LL: cell bodies and fibers over entire ipsilateral extent	probably retrograde vector transport to LL	2C
	#10	2	rAAV2/9	1	1	right	not found, likely more rostral than evaluated	–		
	#11	2	rAAV2/9	2	1	left	not found, likely more rostral than evaluated	–		
	#12	3	rAAV2/9	2	3	left	not found, likely more rostral than evaluated	–		
**On-Target**	#13	10	rAAV2/1	2	3	left	dorsolateral edge of NM	NM: cell bodies and fibers over entire ipsilateral NM, and over parts of contralateral NM; NL: fibers from ipsilateral NM entering entire ipsi- and contralateral NL	retrograde vector transport to contralateral NM; anterograde protein transport along axons terminating in NL	3A; 4A-D; 5A-D
	#14	9	rAAV2/5	2	3	left	lateral of NL, between NL and NA	NM: cell bodies and fibers in ipsi- and contralateral NM; NL: fibers from NM entering ipsi- and contralateral NL, mainly lateral part; NA: fibers and cell bodies in ipsilateral NA; SO: fibers in entire ipsilateral SO, and dorsal contralateral SO; LL: fibers in ipsilateral LL, mainly ventral part; IC: fibers in ipsilateral IC, most likely the core	retrograde vector transport to NM; anterograde protein transport along axons terminating in NL, SO, LL and IC	2D-G; 3B; 4E-H; 5E-H
	#15	11	rAAV2/9	1	2	right	not found, likely close to/inside NL	–		
	#16	11	rAAV2/9	2	2	left	not found, likely close to/inside NL	–		

After injection of vector solution into the brainstem, the barn owls showed no postoperative changes in behavior, food intake or weight.

### 2.3 Data analysis

For evaluation of the rAAV-mediated expression, the brains were extracted after perfusion, trimmed for a cross-sectional plane and cryoprotected in 30% sucrose in PBS for 48 ​h. Sections of 30–50 ​μm thickness were cut using a cryostat (Leica CM 1950), and DAPI staining (1 ​μg/ml, Sigma Aldrich) on floating sections was used as a reference signal for general orientation. The sections were mounted on glass slides and cover slipped with Vectashield (Vector Laboratories) as the mounting medium. For fluorescent detection and imaging, a Nikon epifluorescence microscope (Nikon Eclipse Ni-E and the associated Nikon imaging software NIS Elements AR.4.30.02 64-Bit), as well as a fluorescence slide scanner (Axio Scan Z1, Zeiss, and the associated software ZEISS Efficient Navigation ZEN 2.6 blue edition) were used. Further image processing was carried out with Huygens Essential (Huygens 19.10) and ImageJ (ImageJ 2.0.0-rc43/1.50e).

## 3 Results

We aimed to identify a rAAV serotype for opsin expression in barn owl brainstem neurons, for future optogenetic experiments. Three serotypes (rAAV2/1, rAAV2/5 and rAAV2/9) were evaluated for their transduction efficiency. Different injection volumes and expression times were tested in 16 hemispheres of 11 adult barn owls (aged one to five years), to find the best combination for suitable expression.

### 3.1 Transduction efficiency of different rAAV serotypes in the barn owl brain

**Fig. 2:**
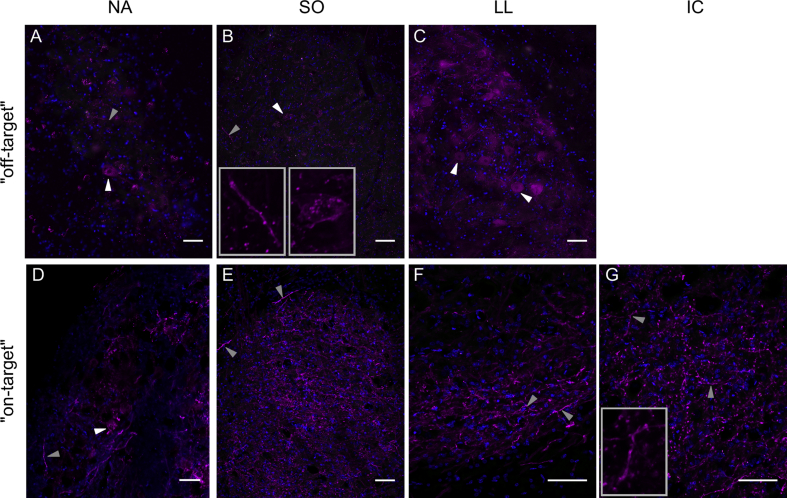
**rAAV2/5 expression after on-/off-target injections**. **1**st **row (A**–**C):** Expression pattern in different brainstem regions following off-target injections far rostral to the target region (border to midbrain; cases #8 and #9 in Table 1). Injection of rAAV2/5 vector resulted in fibers and cell bodies expressing tdTomato within NA (A), SO (B) and LL (C), presumably via retrograde vector transport. **2**nd **row (D**–**G):** Expression pattern in different brainstem regions following an on-target injection very close to NL (case #14 in Table 1). Injection of rAAV2/5 vector resulted in fibers expressing tdTomato within NA (D), SO (E), LL (F) and IC (G), which is plausible via anterograde protein transport. All micrographs are merged images (blue – DAPI, magenta – tdTomato). Some examples of labeled cell bodies are highlighted by white arrowheads, some fibers by gray arrowheads. In addition, both structures highlighted in panel B are also shown in higher-magnification insets; similarly, in panel G, the fiber pointed out by the left-most arrow. Note that many labeled fibers appear as short fragments or even puncta because their course was oblique or perpendicular relative to the sectioning plane. NA – nucleus angularis, SO – superior olive, LL – lateral lemniscus, IC – inferior colliculus. Scale bar 50 ​μm in all panels. For detailed information about injection site, volume and expression time, see Table 1.

The experiments can be divided into two groups. In a first phase, the injection sites were off-target and too far rostral in the brainstem, near the border to cerebellum or midbrain (n ​= ​12 hemispheres). Nonetheless, these experiments provided useful initial basic data on expression efficiency. Both rAAV2/1 and rAAV2/5 resulted in expression, whereas rAAV2/9 did not look promising (overview in Table 1). The rAAV2/1 expression was mainly located around the injection site in the cerebellum, and in the auditory brainstem nuclei NL and NM (Table 1). In contrast, the rAAV2/5 expression was more widely distributed, and additional auditory nuclei expressed the opsin (Fig. 2 1st row; Table 1).

**Fig. 3:**
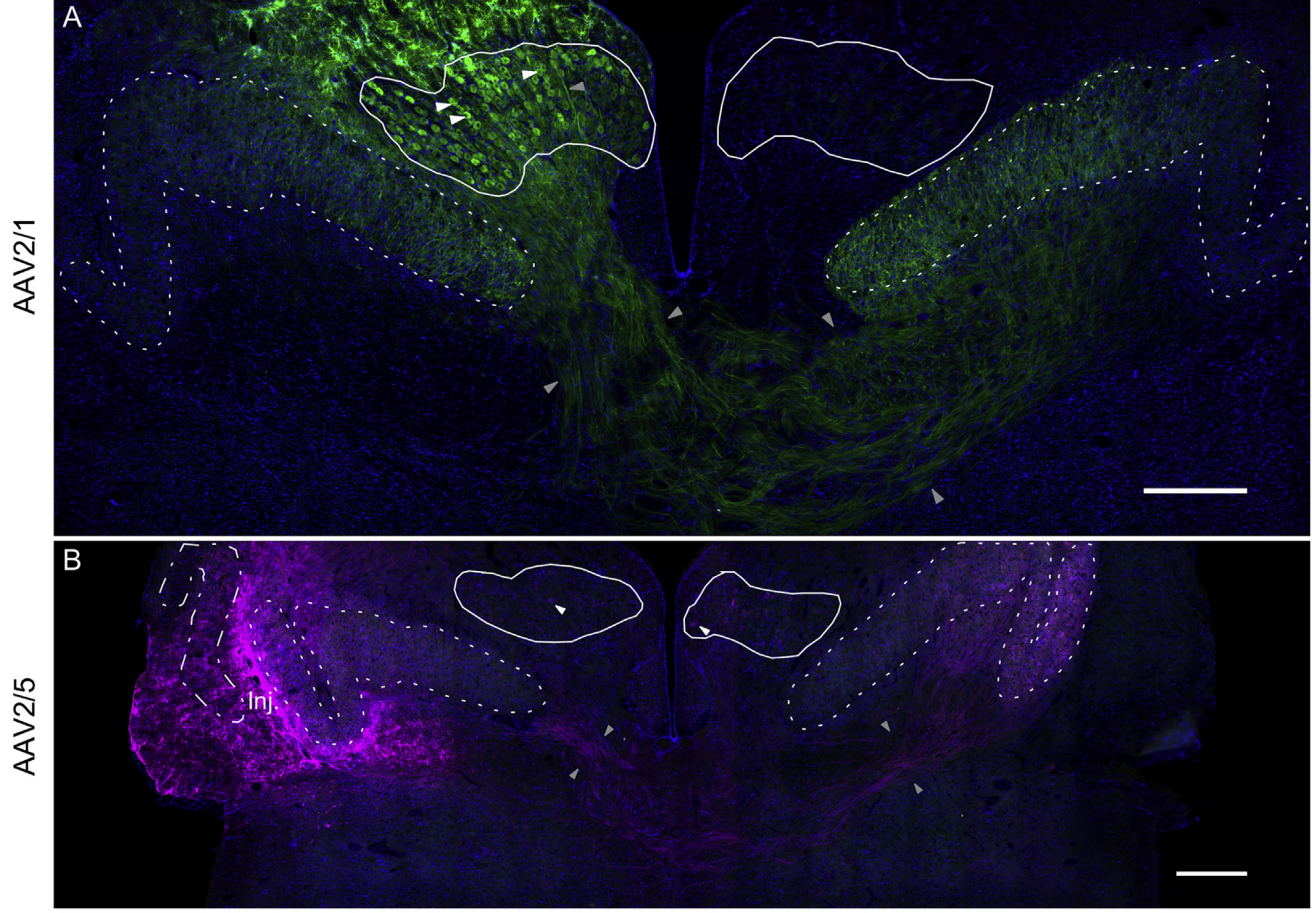
**Overviews of two examples of rAAV2/1 and rAAV2/5 expression**. Both panels show a brainstem slice at a similar rostrocaudal level, at low magnification (scale bars 500 ​μm). The outlines of NM and NL are highlighted in white (NM – solid lines, NL – dotted lines, NA – dot-dashed line). Blue – DAPI, green – GFP, magenta – tdTomato. Some examples of labeled cell bodies are highlighted by white arrowheads, fiber tracts are framed by gray arrowheads. **(A)** An example (case #13 in Table 1) where rAAV2/1 had been injected near the dorsolateral edge of the left NM. Vector presumably spread into NM, leading to expression of GFP in cell bodies of the left NM and fibers projecting from it, towards and into both ipsi- and contralateral NL. **(B)** An example (case #14 in Table 1) where rAAV2/5 had been injected just lateral of the left NL, an area to which many NM axons projecting to NL run, and also both input and output fibers to/from NA. Consequently, a lot of retrograde tdTomato-expression was observed: in the left NA, in both NM, and NM projection axons to and into both NL, including fibers crossing the midline. The injection site is labeled “Inj.” but note that this is always an informed guess, assuming that vector concentration and, consequently, uptake and expression was maximal at the injection site. Depending on the neural candidates for uptake near the injection site, the resulting pattern is not necessarily spherical in appearance.

In a second phase, the injection sites were on-target, within or encroaching on NL and NM in the brainstem (n ​= ​4 hemispheres; example in Fig. 3). Both of the previously promising serotypes – rAAV2/1 and rAAV2/5 – again resulted in strong expression in those auditory nuclei, and both ipsi- and contralateral to the injection site. Additionally, rAAV2/5 was expressed in fibers within higher auditory nuclei (Fig. 2 2nd row; Table 1).

In summary, our results showed effectively transduced neurons in the barn owl brainstem, midbrain and cerebellum with the two serotypes rAAV2/1 and rAAV2/5, but indicated differences in the expression distribution. In contrast, with injection volumes up to 2 ​μl, rAAV2/9 showed no transduction for expression periods up to two weeks in the auditory brainstem (up to 3 weeks when off-target), and may thus be less suitable for gene expression in this bird species.

### 3.2 Axonal transport of serotypes

Solutions injected into the brain diffuse around the injection site. For a volume of 2 ​μl, the average diffusion range has been estimated using dyes to be around 2.4 ​mm in diameter (Myers, 1996). In our on-target injections with 2 ​μl rAAV vector solution, the observed high-density expression areas were around 2 ​mm in diameter. Within this region, uptake of AAV-vector should be highly probable. Gene expression and protein synthesis, however, only take place in the nucleus. Serotype-specific expression patterns can thus be important indicators of different uptake sites and transport efficiencies. For example, anterograde transport will only be evident for the expressed proteins but may show up in labelled axonal tracts and termination sites both near to and far from the transfected neurons, depending on the connectivity. In contrast, expression in cell bodies far distant from the injection site should indicate uptake at synaptic terminals and retrograde axonal transport of the vector towards the cell body. Together, this predicts that vectors that are able to enter neurons at different sites and hitch onto retrograde axonal transport will tend to show a greater spread of expression than those only entering at the soma and/or unable to undergo axonal transport. If transsynaptic migration is also possible (Zingg et al., 2017), anterograde axonal transport of the vector will widen the spatial spread of expression even further.

**Fig. 4:**
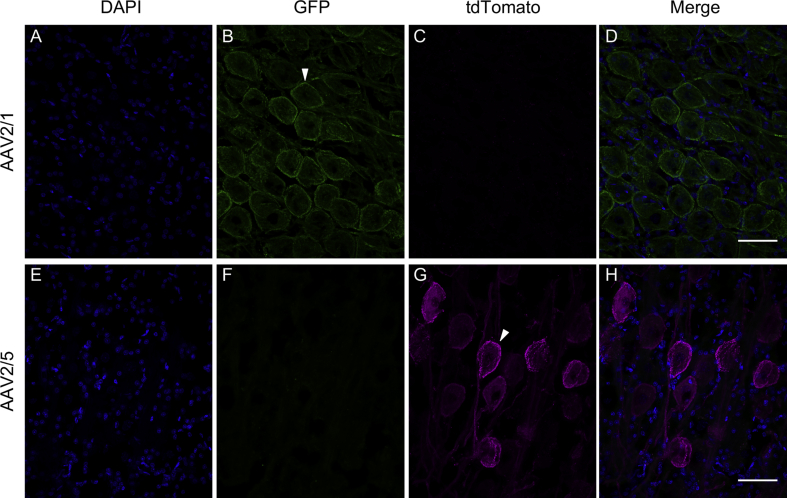
**Examples for the expression of serotypes rAAV2/1 and rAAV2/5 in nucleus magnocellularis**. **A–D:** Expression of GFP, indicating rAAV2/1, in NM cell bodies and fibers, three weeks after injection (case #13 in Table 1). **E–H:** Expression of tdTomato, indicating rAAV2/5, in NM cell bodies and fibers, also three weeks after injection (case #14 in Table 1). Two examples of labeled cell bodies are highlighted by white arrowheads. Note that in each row (A–C and E–G), the first three panels illustrate identical image frames but separately for 3 fluorescence channels; D and H show the corresponding merged images. Scale bars 50 ​μm. For further information about injection site, volume and expression time, see Table 1.

**Fig. 5:**
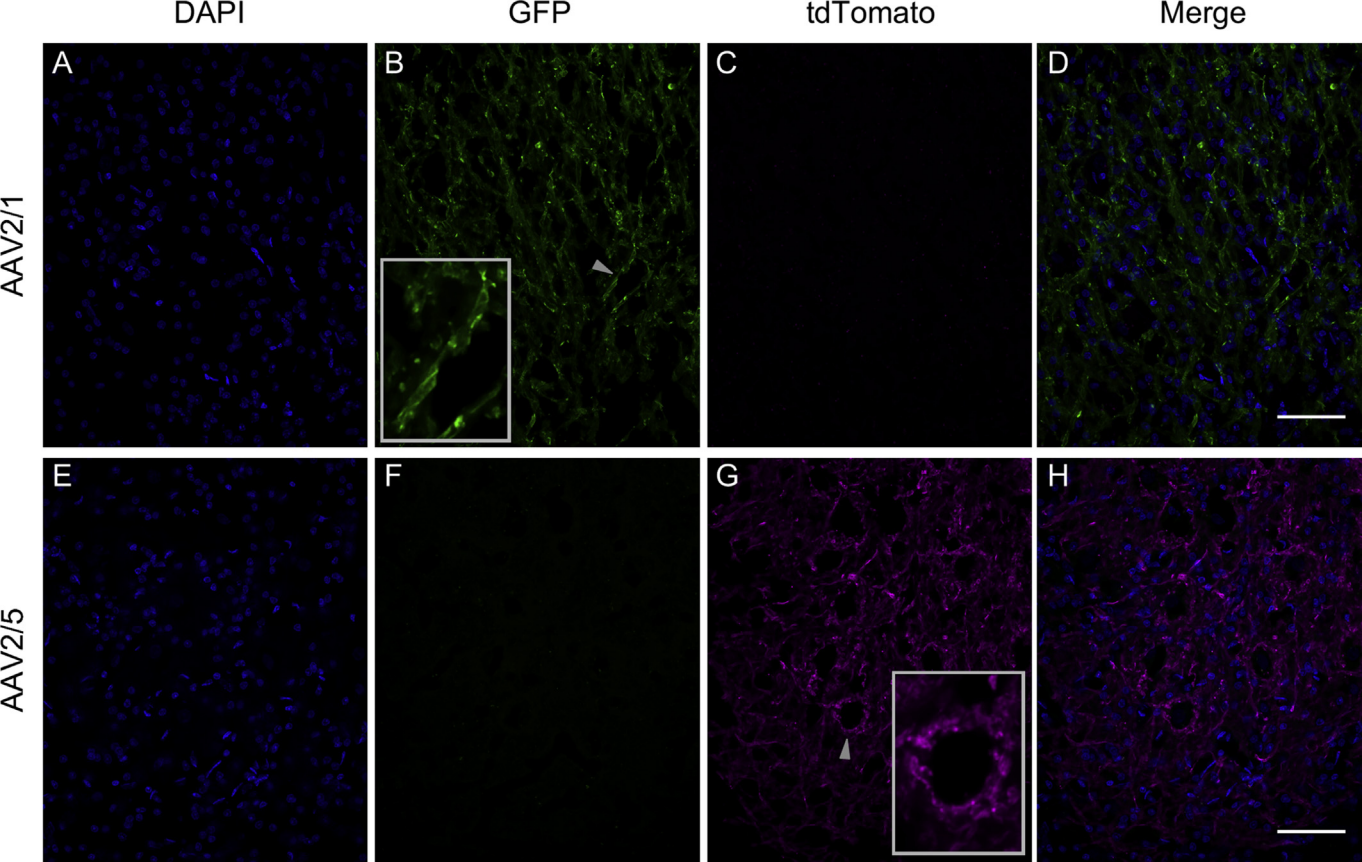
**Examples for the expression of serotypes rAAV2/1 and rAAV2/5 in nucleus laminaris**. **A–D:** Expression of GFP, indicating rAAV2/1, in NM projection fibers ramifying within NL, three weeks after injection (case #13 in Table 1). **E–H:** Expression of tdTomato, indicating rAAV2/5, expressed in NM projection fibers ramifying and terminating within NL, also three weeks after injection (case #14 in Table 1). One example each of a labeled fiber (B) and terminals surrounding a (non-fluorescent) cell body (G) are highlighted by gray arrowheads, and also shown in higher-magnification insets. As in Fig. 2, labeled fibers mostly appear as short fragments because their course was oblique to the sectioning plane. Note also that in each row (A–C and E–G), the first three panels illustrate identical image frames but separately for 3 fluorescence channels; D and H show the corresponding merged images. Scale bars 50 ​μm. For detailed information about injection site, volume and expression time, see Table 1.

Differences in expression pattern between rAAV2/1 and rAAV2/5 in our experiments suggested serotype-specific viral spread. rAAV2/5 injections resulted in strong expression close to the injection site as well as in distant brain regions, whereas rAAV2/1 transduced regions close to the injection site and showed less axonal transport after both on- and off-target injections (overview in Table 1). After on-target injections with rAAV2/1 (e.g. Fig. 3A), retrograde transport of the virus vector to ipsilateral NM cell bodies occurred (Fig. 4A–D) and resulted in fluorescent labelling both in the cell bodies and in the entire axonal projections to ipsi- and contralateral NL (Fig. 5A–D), indicating further anterograde transport of the expressed proteins. Additionally, some virus particles appeared to be taken up at synaptic terminals within ipsilateral NL (where the vector had plausibly spread passively) and were retrogradely transported to contralateral NM cell bodies.

In the case of on-target injections with rAAV2/5 (e.g. Fig. 3B), the same retrograde transport of the virus vector to NM cell bodies was evident through the fluorescently labelled somata (Fig. 4E–H). Also, all axonal projections to ipsi- and contralateral NL were labelled, indicating anterograde transport of the expressed proteins (Fig. 5E–H). In addition, fluorescence was observed in fibers and some cell bodies in ipsilateral nucleus angularis (NA) and in axonal terminals of more distant nuclei, such as the superior olive (SO), lateral lemniscus (LL) and inferior colliculus (IC) (Fig. 2 2nd row). Similarly, rAAV2/5 off-target injections in far rostral brainstem regions, close to the midbrain, resulted in expression in cell bodies of auditory brainstem nuclei, such as NA, SO and LL, and thus indicated long-distance retrograde virus vector transport (Fig. 2 1st row).

In summary, both rAAV2/1 and rAAV2/5 showed evidence for efficient axonal transport of vectors and expressed proteins. In our preparation, rAAV2/5 tended to spread over greater distances than rAAV2/1.

### 3.3 Effects of injection volume and expression time

Possible effects of injection volume and expression times were tested with volumes from 1 to 4 ​μl and waiting times from one to five weeks (Table 1). Although due to restrictions in animal numbers not all possible combinations could be systematically tested, we were able to derive clear recommendations for future optogenetic experiments. For rAAV2/9, increasing the injection volume from 1 to 2 ​μl, or increasing the expression time to up to 3 weeks, appeared to have no benefit, as none of our rAAV2/9 injections resulted in expression. In contrast, an injection volume of 1 ​μl and an expression time of at least three weeks proved sufficient for rAAV2/1 to be expressed in NL and NM. With rAAV2/5, a 4 ​μl injection with one week expression time resulted in strong expression around the injection site, plus some axonal transport. After injecting 2 ​μl, we allowed for at least three weeks expression time, which also resulted in reliable expression and axonal transport. Thus, in our preparation, rAAV2/1 and rAAV2/5 both showed reliable expression after 3 weeks, and rAAV2/5 showed evidence of even faster transduction when the vector volume was increased. Regarding the final transduction area in which expression was observed, our trials included too many other variables (i.e., very different injection sites) to draw any conclusions about a relation to the injected vector volume.

## 4 Discussion

Our experiments demonstrate that reliable expression can be achieved using commercially available rAAV-constructs for gene delivery in a non-standard animal model like the barn owl. Injection of different serotypes results in differential axonal transport and should be chosen according to the requirements of the specific experiment.

### 4.1 Serotype-specific expression efficiency of AAV-vectors in birds

The rAAV transduction efficiency is affected by several parameters, such as animal species, expression time, injection volume and site, and virus titer. Previous experiments highlighted the difficulties of finding a suitable AAV serotype for gene expression in avian brain tissue (Matsui et al., 2012). Matsui et al. achieved no expression with AAV2 in neurons of chickens and zebra finches. Only an avian adeno-associated virus (A3V) showed effective transduction results in the auditory brainstem of embryonic chickens. While AAV2 - in contrast to AAV1, 5, 8 and 9 - shows a desirable preference for infecting neurons rather than glia in brain tissue (Bartlett et al., 1998), the transduction area of AAV2, relative to those other serotypes, is reduced (Watakabe et al., 2015). Suitable pseudotyped AAVs combine the desirable features of two serotypes into a vector of improved transduction efficiency (Burger et al., 2004). In the zebra-finch forebrain, injections of pseudotyped AAV2/1 and 2/5 showed the best results (Heston & White, 2017). Among a greater variety of serotypes and promotors tested, Heston & White (2017) identified rAAV2/1, with a CAG promotor, as their first choice (Heston & White, 2017) This agrees well with our findings in the barn owl brainstem, where the same serotype, with the same promotor, also resulted in the locally densest expression. On the other hand, Roberts et al. (2012) achieved good expression with rAAV2/9 in the zebra-finch forebrain while the same serotype resulted in only poor expression in the tests of Heston & White (2017), and failed completely in our hands in the barn owl. Taken together, the best current recommendation for virus-mediated gene delivery using commercial rAAV vectors in the brains of birds is to start with the pseudotyped rAAV2/1 and 2/5.

### 4.2 Viral spread and its enhancement by axonal transport

Experiments with different AAV serotypes showed antero- and retrograde spread after injections in brain regions in mice (Aschauer et al., 2013; Castle et al., 2014; Cearley & Wolfe, 2007; Rothermel et al., 2013; Zingg et al., 2017) and rats (Castle et al., 2014; McFarland et al., 2009). Even transsynaptic transport of the virus vector is possible (Zingg et al., 2017). In zebra finches, retrograde transport was seen in the forebrain (Heston & White, 2017). Axonal transport could be an explanation for the difference in rAAV2/1- and rAAV2/5-driven fluorescence intensity around the injection site in the barn owl brainstem, despite the similar titer of both virus-vector solutions. Our target injection site is part of the lower auditory pathway in birds, and after rAAV2/5 injection, fluorescent labelling was observed in fibers within higher auditory brainstem and midbrain nuclei, suggesting anterograde transport. Compared to that, injections of rAAV2/1 resulted in the fluorescence being more concentrated around the injection site - only NM cell bodies and NM fibers innervating both NL were labelled - suggesting that fewer expressed proteins were transported away. Serotype-specific transport properties offer opportunities for different experimental designs. The particularly high probability of axonal transport after rAAV2/5 injection may be put to use for achieving expression in different target areas of a specific neural circuit with a single injection. rAAV2/1 on the other hand, is more suitable for experiments that focus on a specific region because it appears to restrict the higher density of expressed protein to the target site. With both vectors, it may be possible to optogenetically activate not only neural somata near the injection site but also their axonal projections in more distant brain regions (Lee et al., 2010; Yizhar et al., 2011).

### 4.3 AAV-mediated expression is expected to remain stable for an extended period of time

Optimal transduction results are determined by an interplay of injection volume, virus titer and time for expression (Zingg et al., 2017). Systematic trials in rat and mouse brains showed a required time interval of two weeks after injection to reach a stable number of expressing neurons (Reimsnider et al., 2007; Zingg et al., 2017). Although less extensive, our trials confirmed similar trends for the barn owl. In general, three weeks expression time were found adequate in our experiments. With rAAV2/5, a volume increase already resulted in good expression after only one week. At the other extreme, our longest tested interval of 5 weeks (with rAAV2/1) still showed good expression. This leads us to expect that expression will remain stable for an extended period of time in owls, as previously demonstrated in mice and rats for at least up to three or nine months, respectively (Reimsnider et al., 2007; Zingg et al., 2017). Furthermore, and encouraging for work with a species of limited availability, such as the owl, the age of the animal seems not to be a limiting factor, as rAAV injections resulted in good transduction efficiency in brains of adult mice and rats (Aschauer et al., 2013; Burger et al., 2004). In summary, stable expression for several months can be reasonably expected in adult barn owls and is an excellent prerequisite for future optogenetic experiments.

## 5 Conclusion

Our study shows that commercially available rAAV vectors are suitable for virus-mediated expression of target genes in the barn-owl brain. The serotypes rAAV2/1 and rAAV2/5 showed reliable expression and can be recommended for future optogenetic experiments. Due to its wide spread, serotype rAAV2/5 provides an interesting option for manipulating several nuclei of the auditory pathway after only one vector injection. In contrast, rAAV2/1 is more suitable for targeted injections whose expression remains confined to a local region.

## Author contributions

C.K. defined the research question and acquired the funds; N.T., C.K. and K.J.H. developed the experimental protocols; N.T. performed all experiments and analyzed the data; N.T. drafted the manuscript; N.T., K.J.H. and C.K. critically revised the manuscript.

## Animal ethics statement

All experiments were approved by the relevant local authorities (permit No. AZ 33.19-42502-04-17/2518).

## Conflict of interest

The authors confirm that there are no conflict of interest to declare.

## Data availability

The original image files used in the figures of this paper, plus additional overview images, are available from the Dryad digital repository, at https://doi.org/10.5061/dryad.9ghx3fffh.

## References

[bib13] Aschauer D.F., Kreuz S., Rumpel S. (2013). Analysis of transduction efficiency, tropism and axonal transport of AAV serotypes 1, 2, 5, 6, 8 and 9 in the mouse brain. Plos One.

[bib36] Bartlett J.S., Samulski R.J., McCown T.J. (1998). Selective and rapid uptake of adeno-associated virus type 2 in brain. Hum. Gene Ther..

[bib1] Boyden E.S., Zhang F., Bamberg E., Nagel G., Deisseroth K. (2005). Millisecond-timescale, genetically targeted optical control of neural activity. Nat. Neurosci..

[bib21] Burger C., Gorbatyuk O.S., Velardo M.J., Peden C.S., Williams P., Zolotukhin S., Reier P.J., Mandel R.J., Muzyczka N. (2004). Recombinant AAV viral vectors pseudotyped with viral capsids from serotypes 1, 2, and 5 display differential efficiency and cell tropism after delivery to different regions of the central nervous system. Mol. Ther..

[bib7] Carr C.E., Boudreau R.E. (1993). Organization of the nucleus magnocellularis and the nucleus laminaris in the barn owl: Encoding and measuring interaural time differences. J. Comp. Neurol..

[bib32] Carr C.E., Konishi M. (1990). A circuit for detection of interaural time differences in the brain stem of the barn owl. J. Neurosci..

[bib5] Carr C.E., Pena J.L. (2016). Cracking an improbable sensory map. J. Exp. Biol..

[bib8] Carr C.E., Shah S., McColgan T., Ashida G., Kuokkanen P.T., Brill S., Kempter R., Wagner H. (2015). Maps of interaural delay in the owl's nucleus laminaris. J. Neurophysiol..

[bib29] Castle M.J., Gershenson Z.T., Giles A.R., Holzbaur E.L., Wolfe J.H. (2014). Adeno-associated virus serotypes 1, 8, and 9 share conserved mechanisms for anterograde and retrograde axonal transport. Hum. Gene Ther..

[bib28] Cearley C.N., Wolfe J.H. (2007). A single injection of an adeno-associated virus vector into nuclei with divergent connections results in widespread vector distribution in the brain and global correction of a neurogenetic disease. J. Neurosci..

[bib23] Fitzsimons H.L., Bland R.J., During M.J. (2002). Promoters and regulatory elements that improve adeno-associated virus transgene expression in the brain. Methods.

[bib10] Goncalves M.A. (2005). Adeno-associated virus: From defective virus to effective vector. Virol. J..

[bib31] Halbert C.L., Lam S.L., Miller A.D. (2007). High-efficiency promoter-dependent transduction by adeno-associated virus type 6 vectors in mouse lung. Hum. Gene Ther..

[bib30] Han X., Chow B.Y., Zhou H., Klapoetke N.C., Chuong A., Rajimehr R., Yang A., Baratta M.V., Winkle J., Desimone R., Boyden E.S. (2011). A high-light sensitivity optical neural silencer: Development and application to optogenetic control of non-human primate cortex. Front. Syst. Neurosci..

[bib20] Heston J.B., White S.A. (2017). To transduce a zebra finch: Interrogating behavioral mechanisms in a model system for speech. J. Comp. Physiol. A Neuroethol. Sens. Neural. Behav. Physiol..

[bib16] Keplinger S., Beiderbeck B., Michalakis S., Biel M., Grothe B., Kunz L. (2018). Optogenetic control of neural circuits in the Mongolian gerbil. Front. Cell Neurosci..

[bib39] Konishi M. (2000). Study of sound localization by owls and its relevance to humans. Comp. Biochem. Physiol. A Mol. Integr. Physiol..

[bib4] Konishi M. (2003). Coding of auditory space. Annu. Rev. Neurosci..

[bib37] Lee J.H., Durand R., Gradinaru V., Zhang F., Goshen I., Kim D.S., Fenno L.E., Ramakrishnan C., Deisseroth K. (2010). Global and local fMRI signals driven by neurons defined optogenetically by type and wiring. Nature.

[bib11] Mastakov M.Y., Baer K., Symes C.W., Leichtlein C.B., Kotin R.M., During M.J. (2002). Immunological aspects of recombinant adeno-associated virus delivery to the mammalian brain. J. Virol..

[bib35] Matsui R., Tanabe Y., Watanabe D. (2012). Avian adeno-associated virus vector efficiently transduces neurons in the embryonic and post-embryonic chicken brain. Plos One.

[bib26] McFarland N.R., Lee J.S., Hyman B.T., McLean P.J. (2009). Comparison of transduction efficiency of recombinant AAV serotypes 1, 2, 5, and 8 in the rat nigrostriatal system. J. Neurochem..

[bib12] Mendoza S.D., El-Shamayleh Y., Horwitz G.D. (2017). AAV-mediated delivery of optogenetic constructs to the macaque brain triggers humoral immune responses. J. Neurophysiol..

[bib33] Myers R.D. (1996). Injection of solutions into cerebral tissue: Relation between volume and diffusion. Physiology & Behavior.

[bib24] Niwa H., Yamamura K., Miyazaki J. (1991). Efficient selection for high-expression transfectants with a novel eukaryotic vector. Gene.

[bib3] Payne R.S. (1971). Acoustic location of prey by barn owls (*Tyto alba*). J. Exp. Biol..

[bib6] Pena J.L., Cazettes F., Beckert M.V., Fischer B.J. (2019). Synthesis of hemispheric ITD tuning from the readout of a neural map: Commonalities of proposed coding schemes in birds and mammals. J. Neurosci..

[bib22] Rabinowitz J.E., Rolling F., Li C., Conrath H., Xiao W., Xiao X., Samulski R.J. (2002). Cross-packaging of a single adeno-associated virus (AAV) type 2 vector genome into multiple AAV serotypes enables transduction with broad specificity. J. Virol..

[bib38] Reimsnider S., Manfredsson F.P., Muzyczka N., Mandel R.J. (2007). Time course of transgene expression after intrastriatal pseudotyped rAAV2/1, rAAV2/2, rAAV2/5, and rAAV2/8 transduction in the rat. Mol. Ther..

[bib19] Roberts T.F., Gobes S.M., Murugan M., Olveczky B.P., Mooney R. (2012). Motor circuits are required to encode a sensory model for imitative learning. Nat. Neurosci..

[bib15] Rockwell H.E., McCurdy V.J., Eaton S.C., Wilson D.U., Johnson A.K., Randle A.N., Bradbury A.M., Gray-Edwards H.L., Baker H.J., Hudson J.A., Cox N.R., Sena-Esteves M., Seyfried T.N., Martin D.R. (2015). AAV-mediated gene delivery in a feline model of Sandhoff disease corrects lysosomal storage in the central nervous system. ASN Neuro.

[bib25] Rothermel M., Brunert D., Zabawa C., Diaz-Quesada M., Wachowiak M. (2013). Transgene expression in target-defined neuron populations mediated by retrograde infection with adeno-associated viral vectors. J. Neurosci..

[bib27] Salegio E.A., Samaranch L., Kells A.P., Mittermeyer G., San Sebastian W., Zhou S., Beyer J., Forsayeth J., Bankiewicz K.S. (2013). Axonal transport of adeno-associated viral vectors is serotype-dependent. Gene Ther.

[bib17] Stieger K., Le Meur G., Lasne F., Weber M., Deschamps J.Y., Nivard D., Mendes-Madeira A., Provost N., Martin L., Moullier P., Rolling F. (2006). Long-term doxycycline-regulated transgene expression in the retina of nonhuman primates following subretinal injection of recombinant AAV vectors. Mol. Ther..

[bib18] Watakabe A., Ohtsuka M., Kinoshita M., Takaji M., Isa K., Mizukami H., Ozawa K., Isa T., Yamamori T. (2015). Comparative analyses of adeno-associated viral vector serotypes 1, 2, 5, 8 and 9 in marmoset, mouse and macaque cerebral cortex. Neurosci. Res..

[bib9] Xiao X., Li J., McCown T.J., Samulski R.J. (1997). Gene transfer by adeno-associated virus vectors into the central nervous system. Exp. Neurol..

[bib2] Yizhar O., Fenno L.E., Davidson T.J., Mogri M., Deisseroth K. (2011). Optogenetics in neural systems. Neuron.

[bib14] Zincarelli C., Soltys S., Rengo G., Rabinowitz J.E. (2008). Analysis of AAV serotypes 1-9 mediated gene expression and tropism in mice after systemic injection. Mol. Ther..

[bib34] Zingg B., Chou X.L., Zhang Z.G., Mesik L., Liang F., Tao H.W., Zhang L.I. (2017). AAV-mediated anterograde transsynaptic tagging: Mapping corticocollicular input-defined neural pathways for defense behaviors. Neuron.

