# Supplementary material for: Gene delivery to neurons in the auditory brainstem of barn owls using standard recombinant adeno-associated virus vectors
Source: Curr Res Neurobiol. 2020 Aug 6;1:100001. doi: 10.1016/j.crneur.2020.100001 (PMC9559881; doi:10.1016/j.crneur.2020.100001)
Supplement: Multimedia component 1 [file mmc1.docx]

*
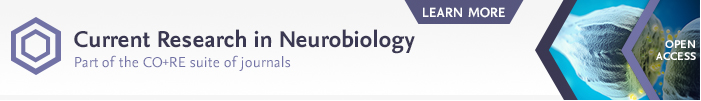
*

**Author Q&A: Gene Delivery to Neurons in the Auditory Brainstem of Barn Owls using Standard Recombinant Adeno-associated Virus Vectors**


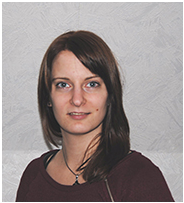


*Nadine Thiele*


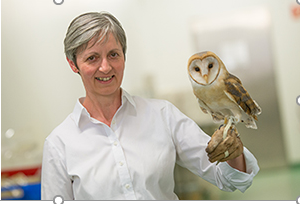


*Christine Köppl
 Both at Department of Neuroscience, School of Medicine and Health Sciences, Carl von Ossietzky University Oldenburg, Germany*

Nadine Thiele and Christine Köppl share their story from research to published article in Current Research in Neurobiology: **‘**[**Gene delivery to neurons in the auditory brainstem of barn owls using standard recombinant adeno-associated virus vectors**](https://www.sciencedirect.com/science/article/pii/S2665945X20300012)**‘**

- Could you tell us a little bit about the basis for your study?

Neuronal activity generates extracellular field potentials, which can be measured non-invasively with electrodes on the surface of the head, but typically represent the activity of several brain areas. A commonly used field potential in auditory neuroscience is the so-called binaural interaction component (BIC). It is represented by several waves and the specific sources of each of these waves are to date still unknown. Our overall aim is to investigate the different sources of binaural information processing and we focus on interaural time differences (ITD), which represent the differences of sound arrival time between the ears and is an important cue for sound localization. In birds, neural ITD processing begins in nucleus laminaris (NL) and we aim to manipulate the neuronal activity of this brain area to study the effect on the BIC. Using optogenetic methods for this task would be hugely advantageous, and the current paper describes the first major step: using different viral vectors to express light-sensitive opsin to enable neuronal inhibition using light.

- How does it help to advance the scientific field to now have these optogenetic tools available for studying the barn owl?

The main advantage of optogenetic manipulation compared to other methods, such as e.g., lesion studies, is its reversibility. Neuronal activity will only be manipulated during the light stimulation and, except for the direct electrode/light fiber tracks, brain tissue remains undamaged. We will be able to make real-time measurements and repeat them over a long time period without significant brain injuries that could affect neuronal circuits in general. The next major step to complete the toolbox is to implement the light stimulation in barn owls and show that it does indeed inhibit our target neurons.

- What were the scientific or other challenges that you faced and how did you overcome them?

In the meantime, optogenetics is a popular method, mostly for experiments using mice, and a lot of different specifications for virus vectors or genetic modifications are known. We aim to implement optogenetics in a non-standard laboratory animal. Therefore, a lot of literature searching and correspondence with different colleagues was needed to draft the first experimental plans; e.g., we contacted different groups that conduct experiments with virus injections in birds to ask for their experiences and recommendations for promising virus vectors. A big advantage was the opportunity to work with Jannis Hildebrandt and his team, who carry out optogenetic experiments with mice. With their help, a promising start was possible and we could immediately limit our selection to AAV viruses. The next challenge was that there was no standard stereotaxic brain atlas for barn owls. We had to adjust our setup for virus injections and needed to establish coordinates for the target regions almost from scratch. Additionally, compared to mice, the reproductive rate of owls is much lower and is a limiting factor for the number of experiments per year. Therefore, we tried to use as few barn owls as possible by injecting in both brain hemispheres and initially without knowing the correct coordinates. The first aim was to see if there was gene expression at all, with any of the vectors on our list. The next challenge was the unknown expression time and, after a few weeks of uncertainty, it was really exciting to analyze the first brain sections.

- Christine and Nadine, readers might be interested in aspects that go beyond the scientific paper published. Is there something about your perseverance individually or as part of a team that you think made it possible to succeed with your research?

In science, and especially when you have to establish a new method, it is really important not to be easily frustrated. Sometimes one has to redo things over and over again until one knows how it works, and this process can easily take weeks or months. In our lab, everyone works on different projects, but all know this learning process and are really supportive and we try to motivate and help each other where we can.

Christine has worked with barn owls for nearly three decades now and says “I consider it a privilege to work with that animal, despite all the small and large difficulties. We have limited control over when and how many animals will be available, as breeding is seasonal. I have learned to plan carefully and be patient.”

- Are there any insights from your perspective that you would like to share with young investigators or those thinking about whether to dedicate their careers to studying the brain?

The brain is a fascinating organ and a lot of working groups try to understand neuronal processes, but it is still not known how everything works and how it is connected. We focus only on one topic (hearing) and, additionally, restrict the project to specific regions. This paper and the following studies focus only on a few nuclei of the whole auditory pathway, mainly on the nucleus laminaris, and it is really extraordinary how complex it is to try to explain one single piece of a one-million piece puzzle.

- What was it like working together as a team on this project and did you or the science benefit from the diversity of perspectives?

As mentioned above, a huge advantage was to have a lab focusing on the field of optogenetics in our department. We had really short communication paths and discussed the intermediate stages until we had a running setup and successful experiments. Furthermore, presenting interim results at conferences and discussions with other researchers were helpful, too. We would encourage young investigators to attend as many conferences as possible, learn from other researchers, and building up a network.

- Do you have advice for young investigators for how they might surmount their personal or scientific challenges?

Never be afraid to ask questions and be open to accept assistance from others. Nobody knows everything.

- Would you encourage more women and individuals from under-represented backgrounds to get involved in neuroscience?

Interestingly, our team members are mainly female and we have now all worked for years in neuroscience. Maybe some women think it would be difficult to manage scientific work and a family, but again our team is a positive example. In the last few years, we welcomed several children to our community.

- Did you take advantage of some of the options (like double blind review)? We’re also implementing a more transparent review process where reviewers, authors and editors would have a chance to opt into having the review process published along with the paper. Would you welcome a change like this to improve the publishing environment?

We welcome more options for the review process and opted for double-blind review for this manuscript. Publication of the review process, together with the paper, will especially benefit the transparency of the journal. For young researchers, it would also be a good opportunity to become more familiar with review processes.

- Do you have any advice for us as a journal aiming to be a more author-supportive journal and to do things differently?

We appreciated the way the Editor clearly indicated the journal’s position regarding points where the reviewers differed. This is very helpful in cases where it is difficult to satisfy everyone.

- Would you be interested in being a part of our advisory or reviewing editor team to help us to do better?

Not at the present time, sorry.
